# *Candida albicans* lumbar spondylodiscitis in an intravenous drug user: a case report

**DOI:** 10.1186/1756-0500-6-529

**Published:** 2013-12-11

**Authors:** Chang-Hua Chen, Wei Liang Chen, Hua-Cheng Yen

**Affiliations:** 1 Division of Infectious Disease, Department of Internal Medicine, Changhua Christian Hospital, Taichung, Taiwan; 2 College of Medicine & Nursing, Hung Kuang University, Taichung, Taiwan; 3 Department of Medical Imaging, Changhua Christian Hospital, Changhua, Taiwan; 4 Department of Neurosurgery, Changhua Christian Hospital, Changhua, Taiwan

**Keywords:** *Candida albicans*, Lumbar spondylodiscitis, Spine, Vertebral osteomyelitis, Fungal infection

## Abstract

**Background:**

Spondylodiscitis leads to debility, and few data exist on *Candida* spondylodiscitis in patients with intravenous drug use.

**Case presentation:**

We present a case of *Candida albicans* lumbar spondylodiscitis in a patient with intravenous drug use. This patient was treated with surgical debridement and 9 months of fluconazole therapy, and the neurological deficits resolved completely. The infection did not recur clinically or radiologically during 9 months of follow-up.

**Conclusion:**

Although *Candida albicans* lumbar spondylodiscitis is rare, *Candida* should be suspected as a causative pathogen in patients with intravenous drug use except for *Staphylococcus aureus, Pseudomonas aeruginosa,* and *Mycobacterium tuberculosis*. As soon as *Candida albicans* lumbar spondylodiscitis is suspected, magnetic resonance imaging and percutaneous biopsy should be performed. Surgical intervention combined with treatment with antifungal medications can successfully eradicate the infection and resolve the neurological deficits.

## Background

Spondylodiscitis is a rare disease accounting for 2–7% of all cases of pyogenic osteomyelitis, with incidence varying from 1 per 100,000/year to 1 per 250,000/year [1,2]. However, the incidence of spondylodiscitis is rising because of longer life expectancy and more patients with chronic debilitating disease, use of immunosuppressive therapy, increasing use of indwelling devices, and spinal surgery [3-5].

The first patient with fungal *Monilia psilosis* osteomyelitis was reported by Connor in 1928 [6], and there was no report of *Candida* osteomyelitis in the literature until 1970 [7]. A study by Gathe *et al*. showed that approximately 60% of cases of *Candida* osteomyelitis occur in the spine [8]. Few data exist on *Candida* infection in patients with intravenous drug use (IVDU) [9-11]. We report on a case of *Candida albicans* lumbar spondylodiscitis (CaLS) in a patient with IVDU, including the clinical presentation, radiological findings, and outcome of combined surgical and medical treatment after 9 months of follow-up.

## Case Presentation

**Figure 1:**
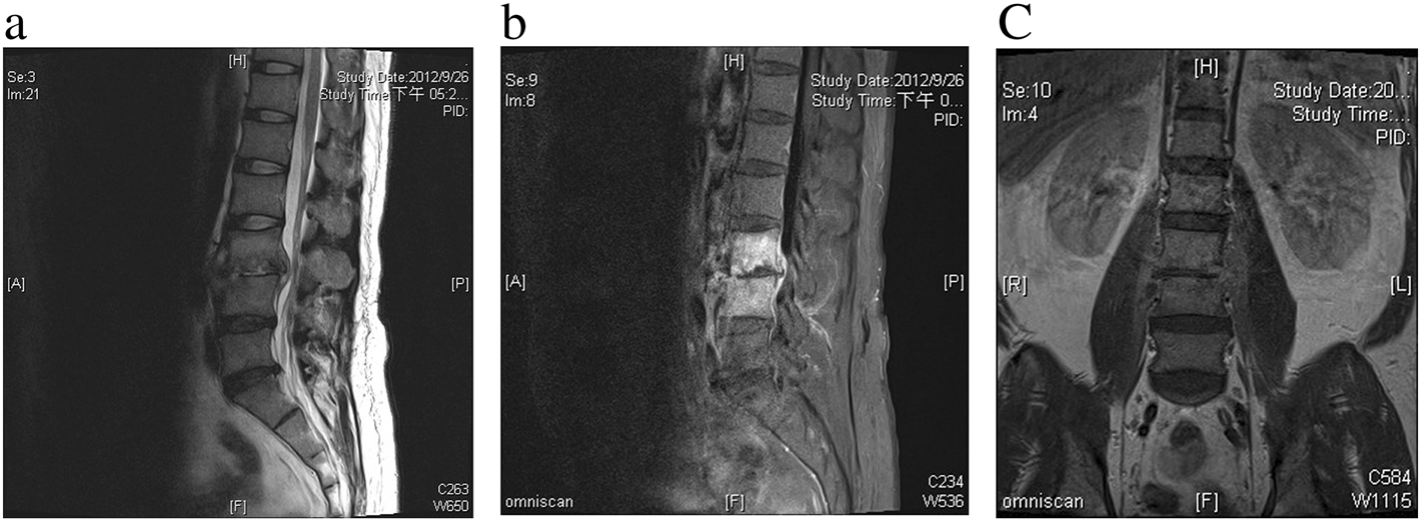
**Magnetic resonance imaging of the lumbar spine showed diffuse bone marrow infiltration plus endplate erosion level at L3 and L4. (a, b, and c)** Magnetic resonance imaging (MRI) of the lumbar spine showed diffuse bone marrow infiltration plus endplate erosion level at L3 and L4 characterized by low signal intensity on the T1-weighted image and high signal intensity on the T2-weighted image, with enhancement in affected bodies and cystic enhanced lesions in epidural and paraspinal regions after administration of gadolinium.

A 41-year-old man with a history of IVDU with injection of heroin initially presented to our clinic with a 3-month history of lower back pain radiating to the lower limbs bilaterally. Findings on neurological examination were normal, and he was treated with oral non-steroidal anti-inflammatory drugs. Three months later, he developed myelopathic symptoms with weakness of both lower limbs and severe back pain and presented to the neurosurgery clinic of our institute. Magnetic resonance imaging (MRI) of the lumbar spine showed diffuse bone marrow infiltration plus endplate erosion level at L3 and L4 characterized by low signal intensity on a T1-weighted image and high signal intensity on a T2-weighted image, with enhancement in affected bodies and cystic enhanced lesions in the epidural and paraspinal regions after administration of gadolinium (Figure 1). Radiologically, pyogenic discitis, vertebral osteomyelitis, and epidural and paraspinal abscesses had to be considered. The patient was treated with oral non-steroidal anti-inflammatory drugs at the clinic. On examination, there was generalized weakness of grade 4 in related muscle groups and positive myelopathic signs in the lower extremities. The initial laboratory findings revealed an elevated erythrocyte sedimentation rate (ESR) (98 mm/hr; normal range, <10 mm/hr) and anemia (12.3 g/dL; normal range, 14–17 g/dL). However, the results of other laboratory tests were within normal limits, including the white blood cell count (12,500 cells/mm^3^).

**Figure 2:**
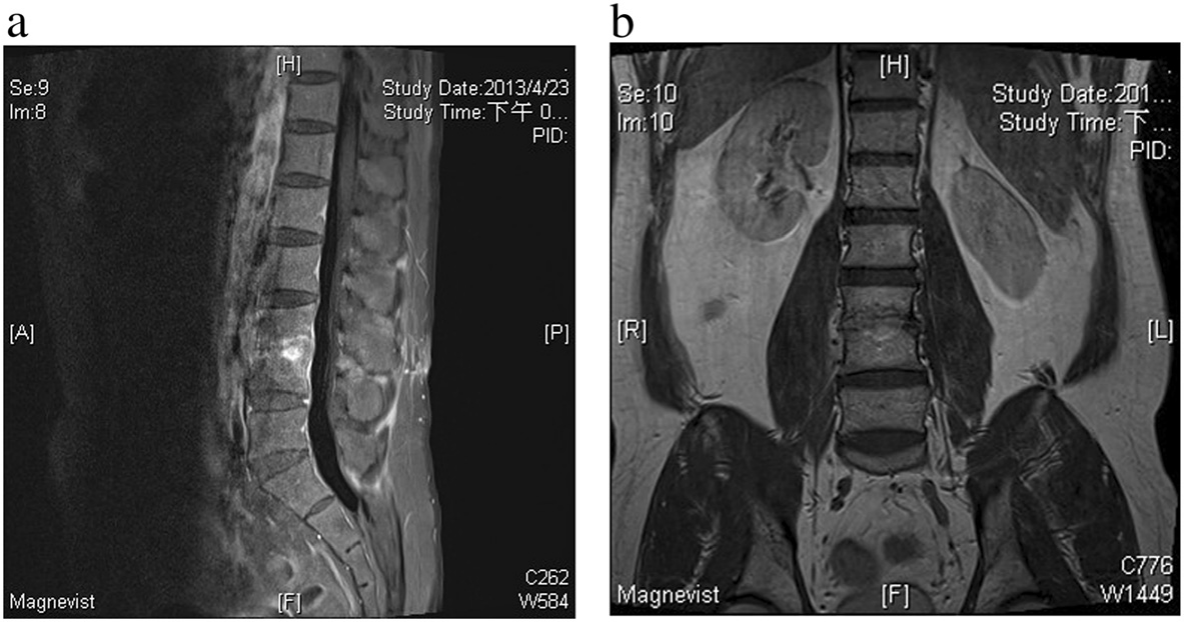
**Magnetic resonance imaging showed significant shrinkage of the vertebral osteomyelitis, discitis, and paraspinal abscesses. (a and b)** Magnetic resonance imaging (MRI) showed significant shrinkage of the vertebral osteomyelitis, discitis, and paraspinal abscesses, although there were still abnormal signals and enhanced lesions in the disc adjacent to the affected vertebral bodies.

Radiography of the lumbar spine revealed spondylosis of the lumbar spine with osteophyte formation and decrease disc height at L3–4. The patient underwent surgery for symptomatic relief; left laminotomy over L3, discectomy over L3–4 under a microscope, and debridement of the disc and endplate were performed and showed necrotic debris and fluid accumulation in the L4–5 disc and endplates as well as a disc bulging over the L3–4 level. The patient was treated empirically with intravenous cefuroxime and gentamycin until candidal infection was confirmed 3 days later. The clinical diagnosis was CaLS, and the patient was subsequently treated with intravenous fluconazole 400 mg/day for 6 weeks and then oral fluconazole 300 mg/day for another 3 months. Nine months later, the patient’s ESR was 8 mm/hr; he had recovered from his neurological deficit with complete return of motor function in the lower limbs and was walking normally, and the motor weakness and pain in his legs had resolved completely. Serial follow-up radiographs after surgery and MRI showed significant shrinkage of the vertebral osteomyelitis, discitis, and paraspinal abscesses (Figure 2), although there were still abnormal signal and enhanced lesions in the disc adjacent to the affected vertebral bodies. The patient recovered very well during the 9-month follow-up period and does not have human immunodeficiency virus infection.

This is the first report of CaLS in a patient with IVDU. Although *Candida* species are low-virulence organisms that inhabit the skin and mucous membrane of humans, the incidence of disseminated and deep-seated *Candida* infections in patients with IVDU is increasing [9-13]. A new pattern of spondylodiscitis in IVDU is emerging, characterized by more frequent unusual pathogen involvement, a severe clinical course, and a need for surgery. Our patient was treated with surgical debridement and antifungal medications.

Because fungal infection has been rarely suggested as a cause of vertebral osteomyelitis [14], we conducted an evidence-based literature review (Table 1) with the keywords “vertebral osteomyelitis”, “fungus”, “spondylodiscitis”, “spine infection”, “fungal spondylitis”, and “fungal spinal osteomyelitis”. Sixty-one patients with fungal vertebral osteomyelitis were reported worldwide (Table 1) [8-13]. Men were more likely to have fungal vertebral osteomyelitis than women, usually around middle age. The most common clinical presentations include local pain, leg weakness, and paraesthesias of the lower extremities. The involved sites of the spine included the cervical spine in two cases, the thoracic spine in seven cases, and the lumbar spine in 52 cases. The pathogens included *Candida albicans* (21), *Candida tropicalis* (6), *Aspergillus fumigatus* (6), *Blastoschizomyces capitatus* (6), *Candida parapsilosis* (5), *Pseudallescheria boydii* (2), *Nattrassia mangiferae* (2), *Rhizopus rhizopodiformis* (2), *Scedosporium apiospermum* (2), *Candida glabrata* (2), *Coccidioides immitis* (1), *Trichosporon asahii* (1), *Scedosporium prolificans* (1), and *Blastoschizomyces dermatitidis* (1). Combined surgical intervention and antifungal therapy has been used since the 1980s; overall, of the 61 patients, five died, two experienced a relapse, and 54 patients recovered well.

**Table 1:** Evidence-based literature review for fungal vertebral osteomyelitis

**Year of publication/country/number of patients/age (y)/sex**	**Clinical symptoms**	**Risk factors**	**Micro-organisms**	**Site of infection**	**Treatment/duration of therapy**	**Clinical outcome/neurological outcome**	**Reference**
1980/Pennsylvania, USA/1/67/F	Backache	Acute myelomonocytic leukemia	*Candida albicans*	Lumbar spine	AmB AmB + 5-FC/NM	Died	Shaikh BS, *et al*. 1980
1985/Kentucky, USA/1/38/M	Leg weakness and paraesthesias	Squamous cell carcinoma of the lung	*Aspergillus fumigatus*	Thoracic spine	AmB + 5-FC/6 wk	Died	Barnwell PA, *et al*. 1985
1982/NM/2/45–61/M	Backache	Previous lumbar laminectomies (2)	*Aspergillus fumigatus* (2)	Lumbar spine (2)	Combination of surgical debridement (2) and antifungal therapy (2) (AmB)/NM	Survived/no ND	Tack KJ, *et al*. 1982
1983/Oregon, USA/3/57–44/M	Backache	Previous L4–5 laminectomy and discectomy	*Candida albicans* (3)	Lumbar spine	A combination of surgical debridement (3) and antifungal therapy (3) (AmB + FLU)/total 12 mo	Survived/no ND	Mawk JR, *et al*. 1983
1987/Washington, USA/1/44/M	Backache	Back trauma and previous spinal surgery	*Candida parapsilosis*	Lumbar spine	A combination of surgical debridement and antifungal therapy (AmB)/NM	Survived/no ND	Friedman BC, *et al*. 1987
1987/Texas, USA/1/64/M	Backache	Back trauma and previous spinal surgery	*Candida parapsilosis*	Lumbar spine	A combination of surgical debridement and antifungal therapy (AmB)/NM	Survived/no ND	Gathe GC, *et al*. 1987
1991/Netherlands/1/46/M	Backache	Previous disc surgery at L4–5 for a herniated nucleus pulposus	*Candida albicans*	Lumbar spine	A combination of surgical debridement and antifungal therapy (ITR)/NM	Survived/no ND, but experienced a relapse 4 wk later	Peters-Christodoulou MN, *et al*. 1991
1994/Italy/1/14/F	Backache	ALL	*Blastoschizomyces capitatus*	Lumbar spine	A combination of surgical debridement and antifungal therapy (AmB + ITR)/2 mo	Survived/no ND	D'Antonio D, *et al*. 1994
1994/Missouri, USA/1/36/F	Backache	Previous cavitary lung infection with *Blastomyces dermatitidis*	*Blastomyces dermatitidis*	Lumbar spine	A combination of surgical debridement and antifungal therapy (AmB to ITR)/40 days to 6 mo	Survived/no ND	Lagging LM, *et al*. 1994
1997/Australia/1/NM/NM	NM	Acute myeloid leukemia	*Pseudallescheria boydii*	NM	Surgical debridement and antifungal therapy	NM	Gatto J, *et al*. 1997
1998/Germany/1/31/M	Backache	None	*Candida tropicalis*	Lumbar spine	AmB/NM	Survived/no ND	Andermahr J, *et al*. 1998
1999/Spain/1/38/M	Backache (5 mo)	ALL	*Blastoschizomyces capitatus*	Lumbar spine	A combination of surgical debridement and antifungal therapy (AmB + FLU)/1 to 5 mo	Survived/no ND	Ortiz AM, *et al*. 1998
1999/Pennsylvania, USA/3/49–54/NM	Upper back pain (1), backache (2)	Recipient of organ transplants	*Candida albicans* (2) *Aspergillus* species (1)	Lumbar spine (2), thoracic spine (1)	A combination of surgical debridement and antifungal therapy (AmB, FLU)/6 wk, 6 mo	Survived/no ND	Williams RL, *et al*. 1999
2001/Taiwan/1/51/M	Neck pain and numbness	Previous urological surgery	*Candida* species	Cervical spine	A combination of surgical debridement and antifungal therapy (AmB, NM)/40 days, 3 mo	Survived/no ND	Wang and Lee 2001
2001/New York, USA/19–64/2 M, 1 F	Backache	After artificial nail use (3)	*Candida albicans* (3)	Lumbar spine (3)	A combination of surgical debridement and antifungal therapy (AmB, FLU)/4–6 wk, 11–12 mo	Survived/1 ND with residual numbness of the right lower extremity	Parry MF, *et al*. 2001
2001/Miami, USA/11/78–28/8 M, 3 F	Omit	DM (2), corticosteroid use (4), chemotherapy for a tumor (1), malnutrition (2)	*Pseudallescheria boydii* (1), *Aspergillus fumigatus* (2), *Candida parapsilosis* (2), *Candida tropicalis* (2), *Coccidioides immitis* (1), *Candida albicans* (3)	Thoracic spine (2), lumbar spine (9)	A combination of surgical debridement and antifungal therapy (AmB base regimen + azole)/6 mo	Died (2/11), survived (9/11)/ND (1/9)	Frazier DD, *et al*. 2001
2002/Ohio, USA/1/NM/NM	Upper back pain ( 4 mo)	None	*Scedosporium apiospermum*	T11–L2	Surgical debridement and antifungal therapy (ITR)/NM	Died	Levine NB, *et al*. 2002
2002/Lebanon/1/65/M	Backache	DM	*Candida albicans*	Thoracic spine, with cord compression	Surgical debridement and antifungal therapy (FLU)/NM	Survived/no ND	El-Zaatari MM, *et al*. 2002
2003/Switzerland/3/NM/NM	Backache	NM	*Candida albicans* (2), *Candida tropicalis* (1)	Lumbar spine (3), 2 with a spinal epidural abscess	Surgical debridement (3) and antifungal therapy (AmB + FLU, AmB alone, AmB + FLU + ITR)	Survived/no ND	Garbino J, *et al*. 2003
2003/Spain/3/72–81/3 M	Backache	DM (2), immunosuppression (2), use of central venous catheters (3), antibiotic use (2), use of parenteral nutrition (2)	*Candida albicans* (1), *Candida glabrata* (1), *Candida parapsilosis* (1)	Lumbar spine	Surgical debridement (3) and antifungal therapy; FLU (2)/NM, AmB + 5-FC (1)/NM	2 cured, 1 relapse	Rodriguez D, *et al*. 2003
2004/Taiwan/1/6/F	Jaw pain	ALL	*Blastoschizomyces capitatus*	Lumbar spine	A combination of surgical debridement and antifungal therapy (ITR)/5 mo	Survived/no ND	Cheung MY, *et al*. 1999
2004/Italy/1/50/F	Backache	L4–5 previous microdiscectomy	*Aspergillus fumigatus*	Lumbar spine	A combination of surgical debridement and antifungal therapy (ITR)/3 mo	Survived/no ND	Lenzi J, *et al*. 2004
2004/Austria/1/62/M	Backache	Recipient of cadaveric kidney transplantation	*Nattrassia mangiferae*	Lumbar spine	A combination of surgical debridement and antifungal therapy (AmB + VOR)/NM	Died	Willinger B, *et al*. 2004
2004/Spain/1/37/M	Backache	ALL	*Blastoschizomyces capitatus*	Lumbar spine	A combination of surgical debridement and antifungal therapy (ITR)/220 days	Survived/no ND	Martino R, *et al*. 2004
2006/Taiwan/1/57/M	Backache	Previous disc puncture and previous radio-frequency nucleoplasty	*Rhizopus rhizopodiformis*	Lumbar spine	A combination of surgical debridement and antifungal therapy (AmB to FLU)/ 8 wk to 3 wk	Survived/no ND; relapse 3 mo later	Chen F, *et al*. 2006
2006/United Kingdom/3/31–70/2 F, 1 M	Backache	Nil	*Candida* species	Cervical spine (1), thoracic spine (1), lumbar spine (1)	A combination of surgical debridement and antifungal therapy (AmB + FLU)/14 days + 3 mo (1), 14 days + 10 wk (1), 12 wk (1)	Survived/no ND	Khazim RM, *et al*. 2006
2008/Turkey/1/63/M	Backache	Colon adenocarcinoma	*Blastoschizomyces capitatus*	Lumbar spine	A combination of surgical debridement and antifungal therapy (AmB to ITR)/40 days to 4 mo	Survived/no ND	Celik AD, *et al*. 2009
2008/Korea/1/42/F	Backache	Previous laminectomy and discectomy	*Trichosporon asahii*	Lumbar spine	A combination of surgical debridement and antifungal therapy (FLU)/5 mo	Survived/no ND	Kim KW, *et al*. 2008
2008/Japan/1/14/M	Backache	Acute myeloid leukemia	*Blastoschizomyces capitatus*	Lumbar spine	A combination of surgical debridement and antifungal therapy (AmB + VOR)/6 mo	Survived/no ND	Yoshihara T, *et al*. 2004
2008/New York, USA/1/43/M	Backache	Near-drowning	*Scedosporium apiospermum*	Lumbar spine	A combination of surgical debridement and antifungal therapy (AmB + VOR, VOR)/40 days to 12 mo	Survived/no ND	Mesfin FB, *et al*. 2008
2009/Spain/1/62/F	Backache	None	*Scedosporium prolificans*	Lumbar spine	A combination of surgical debridement and antifungal therapy (AmB + VOR)/6 wk, 11 mo	Survived/no ND	Garcia-Vidal C, *et al*. 2009
2010/Italy/6/53–74/4 M, 2 F	Omit	Leukemia (1), immunosuppression (1) kidney cancer (1), vesical tumor (1), use of central venous catheters (2)	*Candida albicans* (3), *Candida glabrata* (1), *Candida tropicalis* (2)	Lumbar spine (5), cervical spine (1)	Surgical debridement (1/6), antifungal therapy (6/6) (AmB alone/12 wk then AmB + FLU/12 wk (1); FLU alone/9–36 wk (3); VOR alone/28–33 wk (2)	Survived (6/6)/1 ND (1/6)	D'Agostino C, *et al*. 2010
2013/Taiwan/1/41/M	Backache (2 mo)	None	*Candida albicans* (1)	Lumbar spine	FLU/6 mo	Survived/no ND	This case

*Abbreviations*: ALL = acute lymphoblastic leukemia; AmB = amphotericin B; DM = diabetes mellitus; F = female; 5-FC = 5-fluorocytosine; FLU = fluconazole; ITR = itraconazole; M = male; ND = neurological deficit; NM = no mention in the article; VOR = voriconazole.

There are no non-invasive diagnostic tests or typical radiological findings in CaLS. Reports by Waldvogel and Gathe showed that the delay in diagnosis between the onset of symptoms and the diagnosis of CaLS ranges from 1 month to several years, with an average of 3.3 months [7]. Symptoms such as low-grade fever, malaise, and weight loss are non-specific. Similarly, anemia, neutrophilia, and elevated ESR are also non-specific, representing a chronic inflammatory process, although a markedly elevated ESR is suspicious of infection.

*Candida* spondylodiscitis usually involves the intervertebral disc space with narrowing of the disc cartilage, causing destruction and lysis of the vertebral endplates and underlying vertebral bone [8]. The findings on MRI include an absence of disc hyperintensity and preservation of the internuclear cleft on T2-weighted images in addition to increased signal intensity on gadolinium-enhanced T1-weighted images and early disc destruction and paraspinal abscesses [15]. The delay of antifungal medical or surgical treatment in our case allowed us to evaluate the natural course of the disease. *Candida* affects the spinal segments probably through hematogenous dissemination, and once a focus develops in the vertebral body, it continues an indolent course [12]. In our case, vertebral collapse, psoas abscess formation, and neurological deficits became apparent within 3 months of the onset of symptoms. The reasons for delayed suspicion and diagnosis in our case included a delay in presentation and a delay of investigation. A high index of suspicion and immediate MRI would have confirmed the pathologic lesions before vertebral collapse and neurological compromise developed. As soon as lumbar spondylodiscitis is suspected, MRI and percutaneous biopsy should be performed, followed by medical therapy. This may halt the progression of bony destruction and prevent the need for surgical treatment [16]. Surgery is primarily indicated for failure of or relapse after conservative treatment, significant spinal collapse, and neurological deficits [17].

The management of patients with CaLS includes conservative treatment with biopsy, medical therapy, and bed rest; posterior surgical stabilization and transpedicular biopsy with or without fusion; or anterior debridement of the abscesses, tissue diagnosis, and fusion combined with posterior stabilization. Some investigators have recommended primary reconstruction for all pyogenic infections of the spine to maximize eradication of the infection [18], and others have recommended surgery to aid in diagnosis as well as decompression and stabilization of the spine at the same time [19]. Combined debridement and treatment with an antifungal agent was performed in this case because there is controversy regarding the use of instrumentation in the presence of infection, and in this case the infection was successfully eradicated and the neurological deficits were resolved.

## Conclusions

Although CaLS is rare, *Candida* should be suspected as a causative pathogen in patients with IVDU except for *Staphylococcus aureus, Pseudomonas aeruginosa,* and *Mycobacterium tuberculosis*. Without adequate treatment, the disease is progressive and leads to vertebral destruction and spinal cord and neural compression. Early recognition of CaLS may be difficulty until the patient either develops back pain with symptoms of impending cord compression or develops various grades of neurological deficits. As soon as CaLS is suspected, MRI and percutaneous biopsy should be performed, followed by medical therapy. This may halt the progression of bony destruction and prevent the need for surgery. However, if vertebral collapse and spinal cord compression occurs, surgical intervention combined with antifungal therapy can successfully eradicate the infection and resolve the neurological deficits.

## Consent

Written informed consent was obtained from the patient for publication of this Case Report and any accompanying images. A copy of the written consent is available for review by the Editor-in-Chief of this journal.

## Abbreviations

CaLS: Candida albicans lumbar spondylodiscitis; ESR: Erythrocyte sedimentation rate; IVDU: Intravenous drug use; MRI: Magnetic resonance imaging.

## Competing interests

The authors declare that they have no competing interests.

## Authors’ contributions

Both CHC and HCY managed this patient, and HCY operated for him. WLC interpretated the findings of image. CCH analyzed the data regarding the infectious diseases and wrote the manuscript. All authors read and approved the final manuscript.
